# Assessing the Public Health Economic Loss from PM_2.5_ Pollution in ‘2 + 26’ Cities

**DOI:** 10.3390/ijerph191710647

**Published:** 2022-08-26

**Authors:** Yifeng Wang, Ken Sun, Li Li, Yalin Lei, Sanmang Wu, Yong Jiang, Yanling Xi, Fang Wang, Yanfang Cui

**Affiliations:** 1 School of Economics and Management, China University of Geosciences, Beijing 100083, China; 2 Key Laboratory of Carrying Capacity Assessment for Resource and Environment, Ministry of Natural Resources of the People’s Republic of China, Beijing 100083, China; 3 College of Water Resources, North China University of Water Resources and Electric Power, Zhengzhou 450046, China; 4 State Key Laboratory of Water Resource Protection and Utilization in Coal Mining, Beijing 100011, China; 5 Tianjin Academy of Social Sciences, Tianjin 300191, China; 6 School of Management and Economics, North China University of Water Resources and Electric Power, Zhengzhou 450046, China

**Keywords:** PM_2.5_, exposure-response model, health effect, willingness to pay, ‘2 + 26’ cities

## Abstract

Due to the fast growth of China’s economy, urban atmospheric pollution has become a serious problem affecting the public’s physical and mental health. The ‘2 + 26’ cities, as the Jing-Jin-Ji atmospheric pollution transmission channel, has attracted widespread concern. There were several previous studies on the economic loss of public health caused by PM_2.5_ pollution in ‘2 + 26’ cities. To assess the economic loss caused by PM_2.5_ on human health in ‘2 + 26’ cities, this paper used the exposure-response model, the health effect loss model and willingness to pay method to obtain the economic loss from PM_2.5_ pollution with the latest available data in 2020. It was concluded that, in 2020, the economic loss of ‘2 + 26’ cities from PM_2.5_ was spatially distributed low in the east and high in the west. In addition, it was larger in the southern and northern part, which was smaller in the middle of the region. Based on the conclusions, policy recommendations were put forward.

## 1. Introduction

### 1.1. Background

With the concept of sustainable development gradually gaining popularity, saving resources and protecting the environment have become the consensus. However, due to the fast growth of China’s economy, the consumption of energy has increased and resulted in serious air pollution that has caused great harm to all residents. Jing-Jin-Ji and its surrounding areas are particularly polluted. In order to address these environmental issues, the Ministry of Ecology and Environment formulated the ‘Air Pollution Prevention and Control Work Plan for Jing-Jin-Ji and its surrounding areas in 2017’ and determined that the implementation scope was the air pollution transmission channel of Jing-Jin-Ji, which was namely ‘2 + 26’ cities. The reason why the ‘2 + 26’ cities were chosen in this paper is that, although there are several studies about assessing the health effect loss of different cities, it is still important to consider the regional effect on different cities within since the PM_2.5_ pollution is dynamically transmitted. Thus, according to the Air Pollution Prevention and Control Work Plan for Jing-Jin-Ji and its surrounding areas formulated by Ministry of Ecology and Environment, it is more reasonable to consider the ‘2 + 26’ cities as a whole to discuss the health effect economic loss, for which the paper chose the air pollution transmission channel of Jing-Jin-Ji mentioned in the ‘Plan’, which was namely ‘2 + 26’ cities. The ‘Plan’ claimed that the ‘2 + 26’ cities include Beijing, Tianjin, Shijiazhuang, Tangshan, Langfang, Baoding, Zhangzhou, Hengshui, Xingtai, Handan, Taiyuan, Yangquan, Changzhi, Jincheng, Jinan, Zibo, Jining, Dezhou, Liaocheng, Binzhou, Heze, Zhengzhou, Kaifeng, Anyang, Hebi, Xinxiang, Jiaozuo, and Xiangyang.

Air pollution with PM_2.5_ as the characteristic pollutant has now become the most urgent and prominent environmental problem in China. A large number of epidemiological studies at home and abroad have confirmed that particulate matter is the most harmful air pollutant to human health, and it can cause serious damage to the human respiratory system and cardiovascular system. Due to the small diameter of PM_2.5_, it is easy for it to adsorb heavy metals and microorganisms, which can also break through the barrier and enter cells and blood circulation, which is more likely to cause serious damage to the human respiratory system and cardiopulmonary system. Therefore, PM_2.5_ has caused a great amount of social and economic loss, which not only increased the economic expenditure for medical treatment, but also affected the working ability and time of the labor force, resulting in a reduction in labor output and a negative impact on society as a whole.

### 1.2. Significance

Different from the most urban research, based on the “2017 Air Pollution Prevention and Control Work Plan for Beijing-Tianjin-Hebei and Surrounding Areas”, this paper, formulated by the Ministry of Environmental Protection, determined that the research scope is the Beijing-Tianjin-Hebei air pollution transmission channel area, that is, ‘2 + 26’ cities. The ‘2 + 26’ cities are densely populated, and PM_2.5_ concentrations have long been above the standard. Urban residents have been affected by the air pollution for a long time, so the air pollution problem has received extensive attention. However, there is still a lack of assessments of the health impacts and economic loss of atmospheric particulate pollution across related cities in large areas.

The residents’ health is often plagued by the pollution [1,2]; thus, based on the epidemiological survey data of atmospheric particulate pollutants at home and abroad, this paper used the model of Poisson Regression to analyze the health effects and to assess the economic loss that were caused by PM_2.5_ in ‘2 + 26’ cities. In addition, the comprehensive assessment method could be applied to other places to provide strong data support for solving local environmental problems. The regional comprehensive assessment also provides an important basis for government departments to conduct a cost-benefit analysis of particle pollution prevention and control policies.

### 1.3. Literature Review and Research Gap

Air pollution has become the most urgent and prominent environmental problem in China, among which PM_2.5_ is the main component [3,4]. Many epidemiological research studies at home and abroad considered that PM_2.5_ is more harmful to human health compared to other air pollutants [5,6].

In recent years, European and American countries have conducted a large number of estimation studies on the health hazards of atmospheric particulate pollution, which have a significant impact on population mortality and morbidity [7,8]. Compared to foreign studies, China’s research on particulate pollution started relatively late (Table 1). For example, Wang et al. [9] not only quantified the negative health effects of PM_2.5_ through the exposure-response relationship, but they also established a CGE model in a closing economy to change the labor supply and additional medical expenses to the conduction variables, simulating the exogenous effect of PM_2.5_ pollution on the national economy. It concluded that negative health effects caused the total output loss of the industrial sector to be about CNY 23.97 billion. In addition, Xie et al. [10] combined the CGE model with the pollutant emission model to estimate the economic impact on Jing-Jin-Ji in 2020. It can be concluded that the additional health expenditure could reach CNY 16.62 billion under the setting scenario. The research has great reference significance for the selection of health effect terminals in this paper.

In addition, Wang et al. [19] proposed an economic loss evaluation system that related to health effects to efficiently simulate the characteristic concentrations of PM_2.5_ and estimated economic loss, which indicated that the system could provide new perspectives on the economic loss assessment of health, while it might assist those policymakers in its application in real life. The measures to reduce the public health economic loss were used for reference in this paper.

In conclusion, the applications of this research produced huge economic and social benefits and effectively reduced the public health economic loss, which was also greatly significant and complemented the data on PM_2.5_ pollution loss. However, It could be seen that there was less research on PM_2.5_ in ‘2 + 26’ cities. Due to a lack of data from regional epidemiological studies, the current study was largely limited to a city-scale assessment. Overall, research on air health pollution in China is not mature enough.

### 1.4. The Main Work and the Innovations

**Figure 1:**
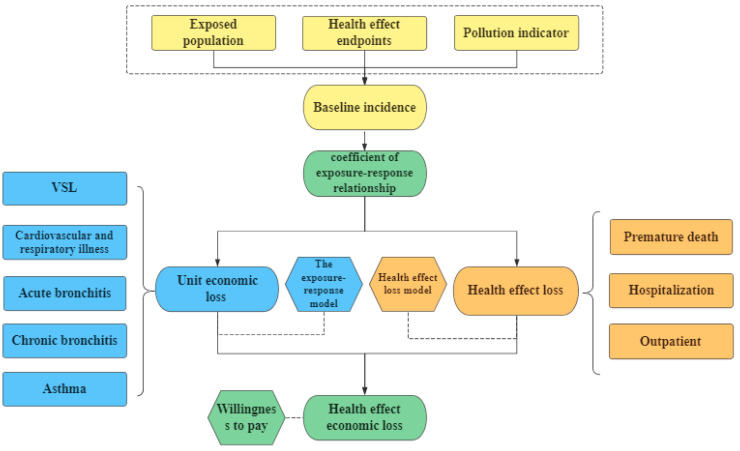
Roadmap.

Therefore, this paper adopted the ambient air quality standard (GB3095-2012), combined with the epidemiological survey data of atmospheric particulate pollutants at home and abroad, based on which the exposure-response model was used to quantitatively analyze the health effects of urban residents in the ‘2 + 26’ cities due to PM_2.5_ pollution and to evaluate the economic loss caused by the health effects of cities in each region (Figure 1). In addition, the determination of ‘2 + 26’ cities refers to the comparison and reference of pollution between different regions and considers the calculation results within the ‘2 + 26’ area by city and disease terminal to achieve a multi-faceted and three-dimensional comprehensive analysis, which was the innovation of this paper.

Overall, this study analyzed the exposure-response relationship and comprehensively assessed the impact on the chosen different health effect terminals, including hospitalization for respiratory diseases and hospitalization for cardiovascular disease, internal medicine and pediatric clinics, chronic bronchitis, acute bronchitis, and asthma. Considering the lack of specific data, there were still other impacts of PM_2.5_ on the residents that were not included in this study.

## 2. Study Area

### 2.1. Background

To solve environmental issues, the Ministry of Ecology and Environment formulated the Air Pollution Prevention and Control Work Plan for Jing-Jin-Ji and its adjacent areas in 2017 and determined that the implementation scope was the air pollution transmission channel of Jing-Jin-Ji, which was namely ‘2 + 26’ cities and included Beijing, Tianjin, Shijiazhuang, Tangshan, Langfang, Baoding, Zhangzhou, Hengshui, Xingtai, Handan, Taiyuan, Yangquan, Changzhi, Jincheng, Jinan, Zibo, Jining, Dezhou, Liaocheng, Binzhou, Heze, Zhengzhou, Kaifeng, Anyang, Hebi, Xinxiang, Jiaozuo, and Xiangyang.

**Figure 2:**
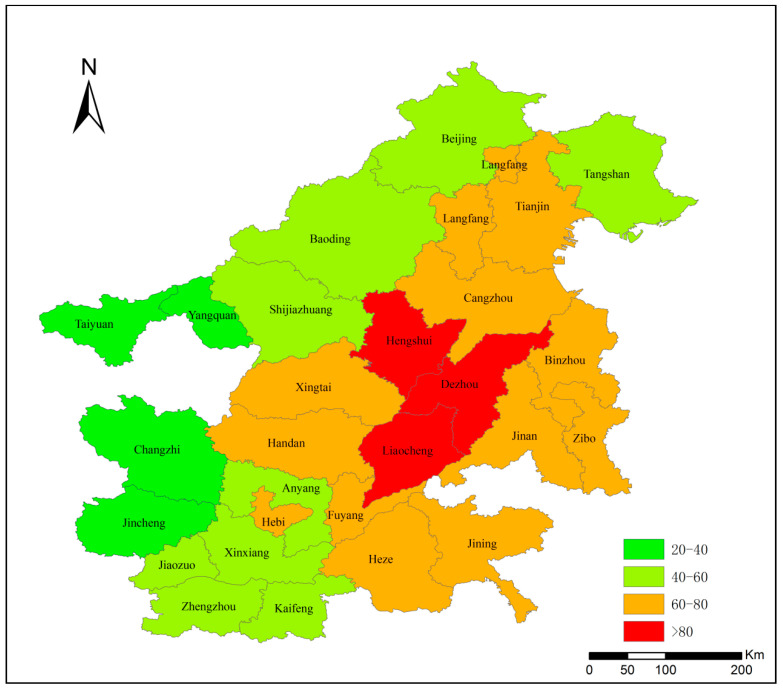
Average PM_2.5_ concentration of ‘2 + 26’ cities in 2020 (μg/m^3^). (Data source: China Environmental Monitoring Station, 2020).

In this paper, the average monitoring concentration data of PM_2.5_ in 2020 were collected by the China Environmental Monitoring Station. Figure 2 revealed that the average annual concentration of PM_2.5_ in ‘2 + 26’ cities exhibited a certain spatial aggregation effect. The three cities of Hengshui, Dezhou, and Liaocheng had the highest average concentrations of PM_2.5_ in 2020. All three cities exceeded 80 μg/m^3^, which revealed that the pollution was still serious. Furthermore, in the marginal area, the air pollution was relatively less serious. PM_2.5_ annual average concentration of Yangquan, Changzhi, Taiyuan, and Jincheng were generally lower than 40 μg/m^3^. It can be seen that the average PM_2.5_ concentration in western cities was lower than that in eastern cities in the ‘2 + 26’ cities area. In general, the PM_2.5_ concentration in ‘2 + 26’ cities shows a radial shape from the center, with a higher concentration in the center of the region and a gradual decrease outward.

### 2.2. Existing Problems

The rapid growth of China’s economy is inseparable from the support of coal resources and the rational use of energy. As one of the core areas, there are still some unreasonable conditions in the development of ‘2 + 26’ cities.

#### 2.2.1. High Pollution Intensity

Although ‘2 + 26’ cities only account for 7.2% of the country’s land area, they consume 33% of the country’s coal. The emission intensity of pollutants per unit area is about four times the national average level, which is the highest air pollution.

#### 2.2.2. Difficulty in Controlling PM_2.5_ Pollution

The operation of some factories, such as those of the chemical industry and those of smelting, and building materials, cannot avoid the production of a large number of polluting solid particles. The existence of these factories in ‘2 + 26’ cities is also a part of social development, and it is difficult to deal with the environmental management of these polluting factories.

At present, many governments in ‘2 + 26’ cities have concentrated the construction of these polluting factories in areas that deviate from the city, but the discharge of harmful gases cannot be avoided; at the same time, in terms of air pollution control methods, government departments need to start from the source of air pollution. Sometimes, a lot of air pollution is generated in the process of control, and a lot of financial resources need to be invested as support.

#### 2.2.3. Air Pollution Control System Needs to Be Improved

Environmental protection is related to the safety of residents’ life; thus, government departments in ‘2 + 26’ cities need to attach great importance to governance since the sources of PM_2.5_ particulate solids are very wide, such as vehicle exhaust emissions, polluting factory emissions, and construction waste generated by the construction industry. These pollutants come from various industries and require coordinated management by various departments.

## 3. Methods and Data

### 3.1. The Exposure-Response Model

Based on the most widely used exposure-response model (Huang and Zhang, 2013), the health risks of the public under PM_2.5_ exposure could be assessed according to the current air pollution epidemiology studies, which is shown in the Equation (1).

(1) $$E=E_{0}\times \exp\left[\beta \left(C-C_{0}\right)\right]$$

where E is the public health effect, which is under the current PM_2.5_ concentration. E_0_ is the health effect under the background concentration of PM_2.5_, β is the coefficient of exposure-response relationship, C is the current concentration of PM_2.5_, and C_0_ is the reference concentration of PM_2.5_.

Based on the reliability and accessibility of the existing data of domestic epidemiology, this paper selected the health effect terminals that were relevant to PM_2.5_, including respiratory disease death, cardiovascular death, acute bronchitis, asthma attack, pediatrics, outpatient, and early death. As a small part of clinical symptoms was difficult to quantify its health effects and it lacked the corresponding statistical data, we only assessed the health effect loss from the selected terminals and their economic loss.

The exposure-response relationship coefficient β is a ratio of change in health benefits from the unit concentration of PM_2.5_ increasing, and it is a terminal for quantitatively evaluating the health hazard pollutants. Here, the estimation of the health effect loss was based on the exposure-response relationship coefficient and the baseline incidence rate. Due to the lack of relevant information in China, this paper selected the conclusions of Kan et al. [20] and used the death exposure-response relationship model as a reference. The coefficients of cardiovascular disease, respiratory system hospitalization, and other exposures were selected from Xie et al. [21]. The data are shown in Table 2.

The baseline incidence of a health terminal indicated the frequency of occurrence of a newly created disease in the health terminal in a particular population over a certain period of time. It was a measure of the impact on the health effects. The benchmark incidence rate of each health terminal in ‘2 + 26’ cities was selected by reference to the health statistics yearbooks, economic statistics yearbooks, and national health service survey report of ‘2 + 26’ cities. As the data of some cities were difficult to obtain, the paper used the average health terminal incidence rate of the national residents as the health baseline data. The data of the exposure-response relationship coefficients and the baseline incidence are demonstrated in Table 2.

### 3.2. Health Effect Loss Model

The health effect loss model is shown in Equation (2):

(2) $$∆E=P\left(E-E_{0}\right)=\mathrm{PE}\left[1-\frac{1}{\exp\left[\beta \left(C-C_{0}\right)\right]}\right]$$

where E is the public health risk under the actual concentration of PM_2.5_; ΔE is the change of public health risk from the variation of the PM_2.5_ concentration; E_0_ is the health effect under the background concentration of PM_2.5_; P is the public quantity (10,000 people); β is the coefficient of the exposure-response relationship (%); C is the current concentration of PM_2.5_; and C_0_ is the background concentration of PM_2.5_.

Compared with the statistical population, each health effect terminal in this paper had a lower occurring probability. While the health effect loss model is more about time series, the statistical poisson distributions are likely to be their actual distribution. The current assessment in accordance with the loss of health effect from PM_2.5_ pollution is on the basis of Poisson regression model in China.

Since the background concentration value was inconclusive, on the basis of the epidemiological survey data of air pollution and the current situation in ‘2 + 26’ cities, this paper selected the baseline concentration value (35 μg/m^3^) in the transition period set by the World Health Organization in 2005 as the background concentration to estimate the health effect loss, which is also the limit of the PM_2.5_ concentration standard adopted by China.

### 3.3. Willingness to Pay Method

The willingness to pay is a method for evaluating the value of environmental quality by measuring the monetary value that the people are willing to pay to improve the health of themselves and others. Based on the results of China’s currently available and reliable research on the willingness to pay method, and combining the unit loss value, this paper assessed the total economic loss caused by the change of the PM_2.5_ concentration. The model is shown in Equation (3):

(3) $$C=\sum ∆E_{i}\times V_{i}$$

where C is the sum of the health loss from PM_2.5_, V_i_ is the unit economic loss of each health effect, and ΔE_i_ is the change of the public death risk and the change of the public morbidity risk from the alteration of PM_2.5_ values.

For premature death from PM_2.5_, the more scientific and complete value of statistical life (VSL) is usually adopted to calculate the economic loss. When using the model, if the study area has a large difference in income levels, the annual average income of the urban population can be selected as the weight. In addition, the VSL of the existing city can be calculated to obtain the unit health loss value of the target city. Similarly, for the determination of the unit economic value of the relevant health effect terminals, the relevant research currently mainly use the disease cost method. The cost of each disease contains the direct medical cost and indirect social cost. The unit economic value of each disease can be calculated by the World Bank in 2007 calculation method and the method mentioned in *Environmental and natural Resources Economics (Tenth Edition)* written by Tietenberg, which contains the human capital law and disease cost method. The VSL is shown in Equation (4):

(4) $$L=L_{1}+L_{2}+L_{3}=S*\sum \left(A_{i}{*P*t}_{i}\right)+S*\sum \left(B_{i}{*P*T}_{i}\right)+S*\sum \left(A_{i}{*P*C}_{i}\right)$$

where L is the value of statistical life according to the World Bank calculation method; L_1_ is the loss of income caused by illness; L_2_ is the income loss caused by premature death; L_3_ is the increase in medical costs caused by illness; S is the per capita national income of the polluted area (CNY/year); P is the population of the polluted area; A_i_ is the incidence of type I disease in the polluted area higher than that in the control area; t_i_ is the per capita loss of working time (years) resulting from the type i disease (recuperation or treatment), which includes the nursing time of non-medical personnel; B_i_ is the mortality rate that is higher in the polluted area than the control area, which was caused by type i disease; T_i_ is the per capita lost working time (years) due to premature death caused by type i disease; C_i_ is the per capita cost of medical care for type i diseases.

As the definition of the sick time of chronic bronchitis was difficult to determine, this paper comprehensively considered that the disease cost method could not be used. The current study generally considered the price of avoiding chronic bronchitis as 32% of its VSL; thus, it was appropriate to use the value as per unit of health loss for chronic bronchitis.

## 4. Results

### 4.1. Unit Economic Value of Each Health Effect Terminal

According to the currently available and reliable unit health economic loss value in China, taking the results of Xie [22] into account and adopting the VSL of Beijing as the benchmark data, this paper calculated each city’s VSL based on the income ratio compared to Beijing. Considering that the scope of the ‘2 + 26’ cities was wide and the income level was quite different, the average annual income of the urban population was selected as the weight. In addition, the VSL in Beijing was assessed to obtain the unit health economic loss value in the ‘2 + 26’ cities. Similarly, for the definition of the economic value unit of respiratory disease hospitalization, cardiovascular hospitalization, acute bronchitis, internal medicine clinics, pediatric clinics, and asthma, the disease cost method was mainly used. The cost conversion of each individual disease contained the direct medical cost conversion and the indirect social cost conversion. This paper used the World Bank’s method in Equation (4) to quantify the unit loss of each chosen health effect terminal. According to the *China Health Statistics Yearbook* in 2020, National Health Service survey data and the ratio of income of urban population in ‘2 + 26’ area in 2020, this paper calculated the unit medical expenses and the loss time of cardiovascular disease, respiratory disease hospitalization, outpatient medical department, and outpatient pediatrics as the unit economic loss value.

**Figure 3:**
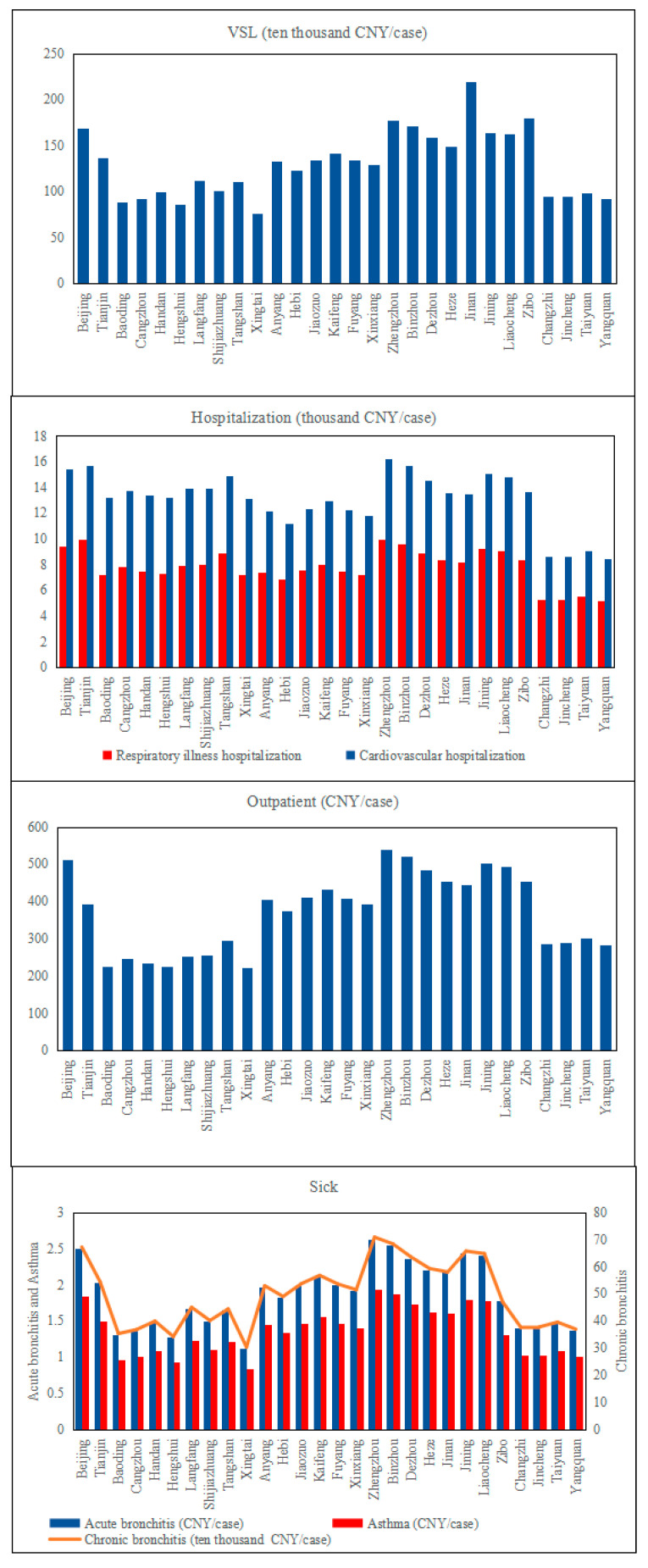
Unit economic loss of each health terminal in ‘2 + 26’ cities.

It can be seen from Figure 3 that, among all the health effect terminals, the unit economic loss of chronic bronchitis accounted for a larger proportion than other health effect terminals, which is followed by cardiovascular hospitalization, premature death (part of the VSL), respiratory illness hospitalization, acute bronchitis, asthma, and outpatient. In addition, it is shown that the unit economic loss of different cities in ‘2 + 26’ cities appears with similar distribution characteristics. Residents in Beijing, Zhengzhou, Binzhou, Jining, and Liaocheng suffer from PM_2.5_ pollution the most, while the unit economic loss of each health effect terminal is lower in Xingtai, Hengshui, and Baoding. In conclusion, the unit economic loss of all the health effect terminals in ‘2 + 26’ cities shows a spatial distribution pattern of high east and low west.

The definition of the suffering time of chronic bronchitis was quite difficult to be determined, so it could not be calculated with the disease cost method. Thus, the paper define that the value of avoiding chronic bronchitis was equivalent to 32% of its VSL, which was taken as the unit loss of chronic bronchitis according to previous research. The unit loss value of acute bronchitis mainly referred to Huang and Zhang [23], and the ratio of the per capita income of urban population in ‘2 + 26’ cities was considered as the coefficient of the unit economic loss of various diseases in 2020. With these data, the unit loss of acute bronchitis and asthma in other cities could be converted. The unit economic loss of chosen terminals in ‘2 + 26’ cities had a certain amplification due to the use of the proportion of income.

### 4.2. Health Effect Loss in ‘2 + 26’ Cities

**Figure 4:**
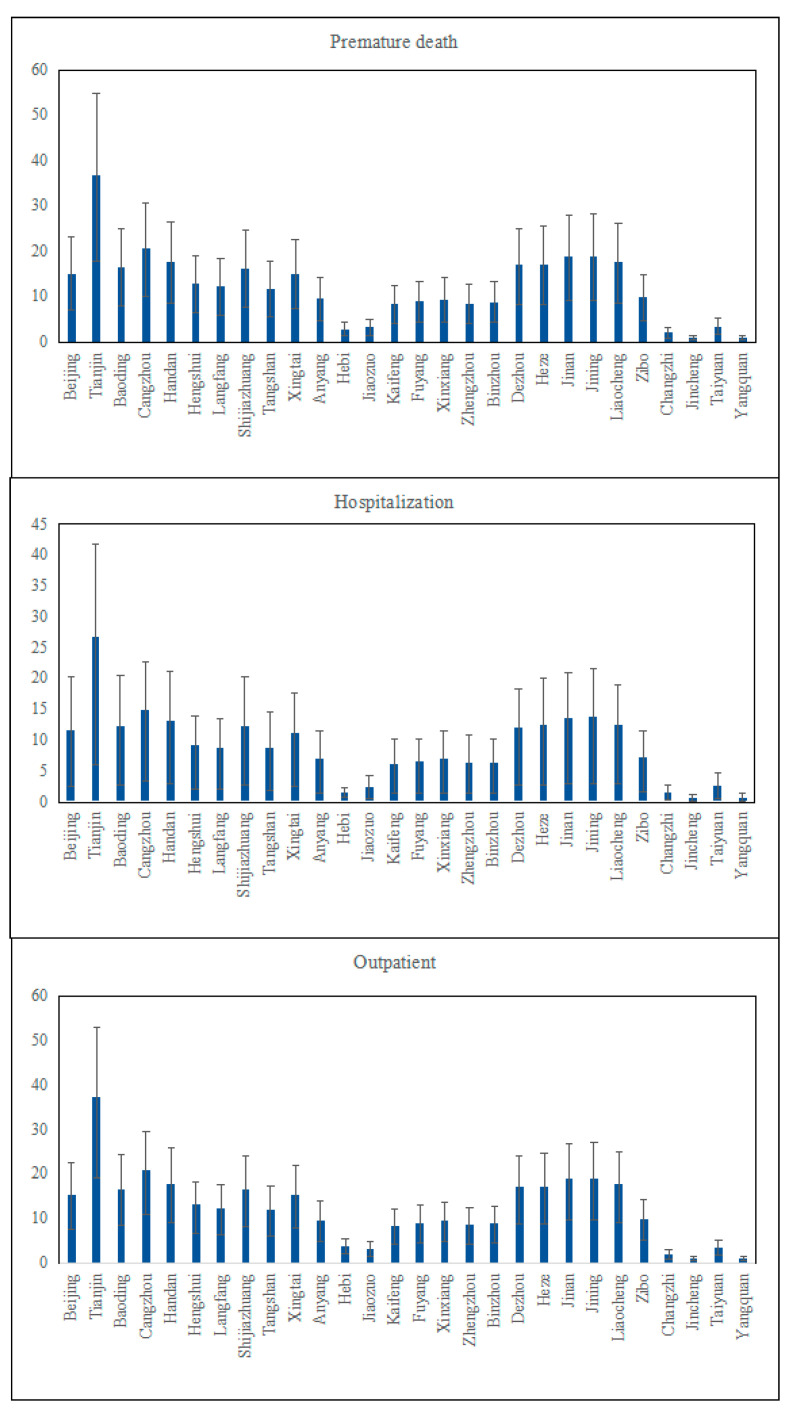
‘2 + 26’ urban public health effect terminal loss values (ten thousand cases).

Due to the different levels of economic development in different cities and the uneven distribution of population density and air pollution within the ‘2 + 26’ cities, the health effect loss caused by PM_2.5_ is also different, showing regional differences. PM_2.5_ exposed the residents to a greater health threat. It can be seen from Figure 4 that different chosen health effect terminals had different sensitivities to PM_2.5_ pollution. The number of the residents affected by PM_2.5_ in ‘2 + 26’ cities was about 9397 (3988, 14,084) thousand. As for premature death, there were about 3412 (1654, 5114) thousand cases of premature death from PM_2.5_, which accounted for the largest proportion compared to other diseases. While the number of cases of chronic bronchitis was 216 (193, 247) thousand, the proportion of chronic bronchitis accounting for the total economic loss was the smallest, which was 216 (193–247) thousand cases. It can be concluded that, among all the negative impacts, the number of outpatient cases was the largest compared to other health effect terminals. The cases of premature deaths due to PM_2.5_ pollution were slightly fewer than those of the outpatient, showing that both two health effect terminals had a similar sensitivity to PM_2.5_ pollution. In addition, the cases of hospitalization also accounted for a great proportion that cannot be neglected.

Tianjin, among all the cities, accounted for the largest proportion of cases compared to other cities to all three health terminals, while Hebi and Jincheng accounted for the smallest. Although the distribution of the PM_2.5_ concentration was uneven within the ‘2 + 26’ cities, the health effect loss did not follow the changes of the PM_2.5_ concentration and presented a spatial distribution in which the loss of those cities in the north-east of the region was large and that in the south-west of the region was low. There might be several reasons accounting for this situation, not only the location of the city might affect the number of cases, but also different environment policies that each city creates might be the core factors.

### 4.3. Total Health Effect Economic Loss in‘2 + 26’ Cities

The amount of economic loss caused by PM_2.5_ in ‘2 + 26’ cities in 2020 was CNY 4650.30 (2259.55, 6950.09) billion, accounting for 4.59% (2.22%, 6.86%) of the GDP.

#### 4.3.1. The Economic Loss of Three Kinds of Health Terminals

**Figure 5:**
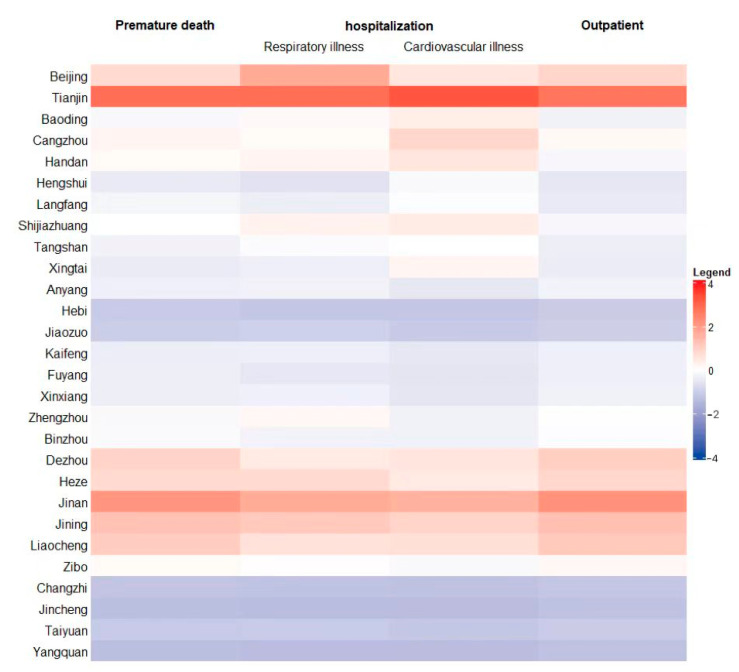
Normalized economic loss of three kinds of health effect terminals in 2020.

It can be seen from Figure 5 that the sensitivity of each health effect terminal to the PM_2.5_ concentration was different. The economic loss caused by premature death caused by PM_2.5_ was about CNY 4560 (2210, 6840) trillion; the loss caused by hospitalization for respiratory diseases was about CNY 644.4 (329.6, 6838.07) billion; the loss caused by hospitalization for cardiovascular disease was about CNY 644.4 (329.6, 6838.07) billion; the loss caused by the outpatient was about CNY 12.27 (8.06, 16.18) billion.

From the comparison between each health terminal selected in this paper, we found that the economic loss from premature death accounted for the biggest proportion, which was 98.1% in the total loss, followed by chronic bronchitis (sick), respiratory and cardiovascular diseases (hospitalization), internal medicine (outpatient), asthma (sick), and pediatrics (outpatient).

In addition, it was found that premature death caused by PM_2.5_ pollution caused most of the economic loss. This is explained not only by the personal lost time but also from the way of determining VSL, which included the ratio of urban personal income. This amplified the economic loss of premature death to a certain extent, making it a bigger proportion in the total health effect economic loss, which also led to a larger proportion of GDP in 2020. Although the health effect economic loss of pediatric outpatient accounted for the smallest proportion, which was CNY 432 million, accounting for 0.02% of the total economic loss, due to the huge calculation base, the actual economic loss caused by it turned out to be an important part that cannot be ignored.

#### 4.3.2. Total Economic Loss

**Figure 6:**
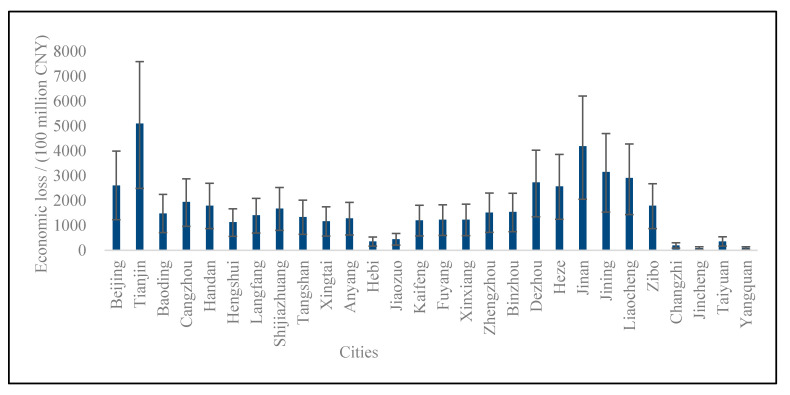
Economic loss of ‘2 + 26’ cities in 2020.

As the economic growth level of each city was different, and the population intensity and air pollution degree were unevenly distributed within ‘2 + 26’ cities, the economic loss from PM_2.5_ was also different. It is revealed in Figure 6 that the loss of Tianjin, Jinan, and Jining were the largest, CNY 509.764 billion, CNY 481.83 billion, and CNY 314.84 billion, respectively, with a total economic loss of CNY 1244.44 billion, accounting for 26.7% of the total economic loss of ‘2 + 26’ cities in 2020. On the contrary, Jincheng and Yangquan in the Shanxi Province accounted for the lowest proportions of the health effect economic loss, which were both less than CNY 10 billion, CNY 9.145 billion, and CNY 9.605 billion, respectively. According to the ratio of the economic loss to GDP in each city, Baoding was the largest, with 8.62%, followed by Cangzhou, with 6.80%. The smallest were in Jincheng and Yangquan, which were 0.87% and 0.69%, respectively. Overall, the health effect economic loss was small in the east and large in the west, with larger loss in the north and south but smaller loss in the middle of the ‘2 + 26’ region.

Considering the geographical location of Jincheng and Yangquan, the proportion of energy and other industries related to PM_2.5_ in the urban industrial structure is smaller than that of other cities. At the same time, due to factors such as heating in the northern district, the economic losses caused by PM_2.5_ pollution in central and northern cities were larger than those in southern cities. In addition, the health effect economic losses of Beijing, Tianjin, Jinan, and Jining were above the average economic loss of all the ‘2 + 26’ cities, which could be affected by the larger exposed population and smaller core city area. On the contrary, the economic losses of Jiaozuo, Hebi, Taiyuan, Changzhi, Jincheng, and Yangquan were all smaller than CNY 100 billion, which implied that, other than geographical location, regional emissions policies also play a significant role in the process of the health effect economic loss. The proportion of health economic loss in the GDP of each city in ‘2 + 26’ cities can be ranked as Baoding, Cangzhou, Tianjin, Jinan, Jining, Liaocheng, Dezhou, Beijing, Heze, Handan, Zibo, Shijiazhuang, Binzhou, Zhengzhou, Langfang, Tangshan, Anyang, Xinxiang, Puyang, Kaifeng, Xingtai, Hengshui, Jiaozuo, Hebi, Taiyuan, Changzhi, Jincheng, and Yangquan.

## 5. Conclusions

### 5.1. Health Effect Loss Cannot Be Neglected

Among all the diseases, premature death, acute bronchitis, and asthma accounted for more than 80% of the total health effect economic loss. It can be concluded that different chosen health effect terminals had different sensitivities to PM_2.5_ pollution. In addition, the health effect economic loss of outpatient was the largest compared to other health effect terminals. The economic loss of premature death due to PM_2.5_ pollution was slightly smaller than that of outpatient, showing that both health effect terminals had similar sensitivities to PM_2.5_ pollution. In addition, the health effect economic loss of hospitalization also accounted for a great proportion that cannot be neglected.

### 5.2. The Economic Loss of Public Health Effect Presents Regional Differences

According to the results above, the economic loss of the public health effect in 2020 accounted for a non-negligible proportion of the GDP, and it presented geographical differences. It was concluded that the health effect economic loss was small in the east and large in the west, with larger loss in the north and south but smaller loss in the middle of the ‘2 + 26’ region. Inside the ‘2 + 26’ cities region, the economic loss from inland cities was smaller than that in other regions. The economic loss in some coastal cities were larger, which accounted for a large proportion. Tianjin, Jinan, and Jining had the biggest health economic losses while Jincheng and Yangquan accounted for the lowest proportion of health effect economic losses. This might be because Tianjin, Jinan, and Jining had more cases of premature death and a larger population. In addition, the lower PM_2.5_ concentration was inseparable from the smaller city core area, which resulted from the local government’s contribution to the reasonable land planning. Not only had it set up corresponding emission reduction policies, but it also made efforts in green public construction to achieve pollution reduction effects.

## 6. Policies

### 6.1. Continue to Implement Coal Consumption Control to Reduce Health Effect Loss

Overall, more attention should be paid to the health effect terminals that are closely related to the public health caused by PM_2.5_ pollution, especially premature death and outpatient. In general, these health effect terminals are generated from PM_2.5_ pollution, which consumes large proportions of coal and energy. Fortunately, the energy consumption structure of ‘2 + 26’ cities began to develop towards diversification. Compared with the national standard value, the percentage of coal consumption in total energy consumption was still large, while the proportion of clean energy was insufficient. For the northern and southern cities in the ‘2 + 26’ area, they need to continue to implement coal consumption control measures to reduce PM_2.5_ emissions. In addition, new air quality standards need to be revised to adapt to the different coal consuming policies. Only the establishment of strict air quality standards can truly protect the health of residents. In this case, air quality standards should be formulated more strictly.

### 6.2. Control the Level of Urbanization and Optimize the Arrangement of the Core Area

The population of the city appears to be one of the influencing factors of PM_2.5_ pollution. Normally, the number of residents is connected to the cases of premature death, respiratory disease, hospitalization and all the other chosen health effect terminals. With a larger population, the city tends to have more economic loss caused by air pollutants, especially PM_2.5_. People in these cities are more easily affected by the pollution generated by more permanent residents, in which case different cities need to take different actions to find out the most suitable population for its own in order to reduce the loss and prevent all the possible problems caused by overpopulation. Cities’ permanent population can be controlled through reasonable policy settings in order to slow down the degree of urbanization, which might lead to the minimization of PM_2.5_ pollution from overpopulation.

In addition, each city needs to determine its reasonable core area based on its different urban population, functions, and positioning to control PM_2.5_ pollution. At the same time, reasonable policy arrangements and government supervision also play a vital role in reducing the PM_2.5_ pollution concentration. At present, some ‘2 + 26’ cities have implemented measures to strengthen the management of single and double numbers of motor vehicles, traffic publicity guidance, and inspection of control of restricted vehicles. These policies have produced significant emission reduction effects.

## Figures and Tables

**Table 1:** Research review.

	Authors	Year	Work
1	Wong et al.	2001 [11]	Utilized the Poisson Regression model and assessed the relationship between the hospital acceptance rates everyday and the representative concentrations of pollutants in Hong Kong and London.
2	Xu et al.	2014 [12]	Established an assessment system on health risk using the epidemiological method and adjusted the heat environment and eliminated health risks of PM_10_ by controlling the intensity of the urban heat island effect.
3	Xie et al.	2014 [13]	Used the Poisson Regression model and the estimating method of environmental value to evaluate the risk of acute health damage to high-concentration PM_2.5_ exposure in Beijing’s residents.
4	Etchie et al.	2017 [14]	Assessed the health and economic loss of Nagpur region. The study utilized a life-table approach to calculate the number of premature deaths and disability-adjusted life years associated with the five health effect terminals associated with PM_2.5_ exposure.
5	Han	2019 [15]	Comprehensively evaluated the health benefits from M_10_ and PM_2.5_ pollution in Zhengzhou from 2014 to 2016.
6	Zeng et al.	2019 [16]	Used spatial interpolation and Ben-map tools to figure out the health loss in China from air pollution, especially PM_2.5_ in 2017, which spatially analyzed the health economic loss at a city scale.
7	Yao et al.	2020 [17]	Calculated the health loss from PM_2.5_ with the log-linear model along with the exposure-response function.
8	Zhang and Cao	2022 [18]	Evaluated the policy effects of air pollution control using a double-difference model (DID).

**Table 2:** The coefficients of the PM_2.5_ exposure-response relationship and the baseline incidence at different health terminals.

Healthy Terminal		β (%)	E Value (‰)
Death	Total mortality	0.40 (0.19, 0.62)	0.0161644
	Respiratory disease mortality	1.43 (0.85, 2.01)	0.0017025
	Cardiovascular mortality	0.53 (0.15, 0.9)	0.007523
Hospitalization	Respiratory diseases	1.09 (0, 2.21)	0.0350411
	Cardiovascular diseases	0.68 (0.43, 0.93)	0.0270904
Outpatient	Pediatrics (0–14 years old)	0.56 (0.2, 0.9)	0.4191781
	Internal medicine (at least 15 years old)	0.49 (0.27, 0.7)	1.1261644
Sick	Acute bronchi	7.90 (2.7, 13)	0.1041096
	Asthma	2.10 (1.45, 2.74)	0.1536986

Source: Kan et al. [20]; Xie et al. [21].

## Data Availability

The datasets used and analysed during the current study are available from the corresponding author on reasonable request.
